# Oxidative Stress: The Hidden Catalyst Fueling Atherosclerosis and Cardiovascular Disease

**DOI:** 10.3390/antiox13091089

**Published:** 2024-09-06

**Authors:** Lorenzo Loffredo, Roberto Carnevale

**Affiliations:** 1 Department of Clinical Internal, Anesthesiological and Cardiovascular Sciences, Sapienza University of Rome, 00161 Rome, Italy; 2 Department of Medical-Surgical Sciences and Biotechnologies, Sapienza University of Rome, 04100 Latina, Italy; roberto.carnevale@uniroma1.it; 3 IRCCS Neuromed, 86077 Pozzilli, Italy

## 1. Introduction

Atherosclerosis is a pathological condition characterized by the inflammation of arterial vessels, leading to serious cardiovascular outcomes such as myocardial infarction, stroke, and death [1]. It is a multifactorial disease driven by classic cardiovascular risk factors, including active or passive smoking [2], dyslipidemia [3], obesity [4,5], hypertension [6], and diabetes [4,7]. These factors contribute to endothelial dysfunction and the development of atherosclerosis primarily through mechanisms involving oxidative stress [8].

Oxidative stress refers to an imbalance between the production of reactive oxygen species (ROS) and the body’s ability to neutralize these reactive molecules using antioxidants [9]. ROS are highly reactive molecules derived from oxygen, including free radicals like superoxide anions and non-radical species like hydrogen peroxide [9]. While ROS play essential roles in normal cellular functions, excessive ROS production or inadequate antioxidant defenses can lead to cellular and tissue damage. This damage is pivotal in the progression of atherosclerosis, as the oxidation of low-density lipoprotein (LDL), endothelial dysfunction, and inflammation all contribute to the formation and progression of atherosclerotic plaques.

Several enzymes, including nicotinamide adenine dinucleotide phosphate (NADPH) oxidase [2,10], myeloperoxidase (MPO) [11], and uncoupled nitric oxide synthase (NOS) [12], are involved in generating ROS, which in turn contribute to a pro-inflammatory state. This pro-inflammatory environment promotes atherosclerosis through mechanisms such as endothelial dysfunction [13], LDL oxidation, diminished antioxidant defenses, and an increased tendency toward a prothrombotic state [14,15].

This Special Issue seeks to explore the role of oxidative stress at various stages of the atherosclerosis process and potential therapeutic approaches to modulate this pathogenic pathway. Understanding the mechanisms by which oxidative stress influences atherosclerosis could pave the way for novel therapeutic strategies aimed at reducing cardiovascular risk.

## 2. Overview of Published Articles

Ballester-Servera et al. explored the role of Lysyl oxidase (LOX)-mediated extracellular matrix in atherosclerosis and aortic valve disease. Their data showed that LOX critically contributes to oxidative stress, as evidenced by high 8-oxo-dG immunostaining, in cardiovascular calcification (Contribution 1, https://doi.org/10.3390/antiox13050523, accessed on 26 April 2024). In line with the importance of oxidative stress in the atherosclerotic process, one of the articles describes, for the first time, the pathogenic role of IL-33 in patients with advanced atherosclerosis (aAT). Specifically, IL-33-primed NETs further induced macrophage activation via the NLRP3 inflammasome, facilitating the release of atherogenic inflammatory mediators and MMPs (Contribution 2, https://doi.org/10.3390/antiox11122343, accessed on 26 November 2022).

In addition, Marquès et al. (Contribution 3, https://doi.org/10.3390/antiox11112147, accessed on 29 October 2022) observed that NOX5 overexpression may favor endothelial dysfunction and contribute to the onset of cardiovascular diseases such as atherothrombosis or stroke by promoting apoptosis, mitochondrial dysfunction, and cytoskeleton changes.

The Special Issue also highlighted the importance of modulating oxidative stress to reduce the atherosclerotic process. For example, Vyas et al. (Contribution 4, https://doi.org/10.3390/antiox12050997, accessed on 25 April 2023) demonstrated the significance of carbon monoxide (CO) in atherogenic manifestations. The authors showed that CO-releasing molecule A1 (CORM-A1), an organometallic compound with a boron core that facilitates the slow and controlled release of CO, improved the histoarchitecture of the thoracic aorta and the serum lipid profile of atherogenic SD rats. They reported that CORM-A1 ameliorated pro-atherogenic manifestations by mitigating miR-34a-5p and subsequently improving mitochondrial biogenesis and cellular redox status.

Finally, the Special Issue presented an important review that aims to describe the current evidence regarding the antioxidant effects of oral antithrombotic therapies in patients with atherosclerotic disease and atrial fibrillation (Contribution 5, https://doi.org/10.3390/antiox12061185, accessed on 30 May 2023). In summary, the review underscores the importance of oxidative stress in the pathophysiology of coronary artery disease (CAD), peripheral artery disease, venous thrombosis, and atrial fibrillation, as well as the pleiotropic antioxidant effects of both oral antiplatelet and anticoagulant therapies. In the clinical setting, the beneficial effects of aspirin, clopidogrel, ticagrelor, and rivaroxaban on oxidative stress have been demonstrated in preliminary observational clinical studies involving patients with coronary artery disease. The authors conclude by stating that the choice of oral antithrombotic therapy based on its antioxidant properties should follow a patient-centered approach, and that in the future, the use of oxidative stress biomarkers could help identify these patients.

## 3. Conclusions

**Figure 1:**
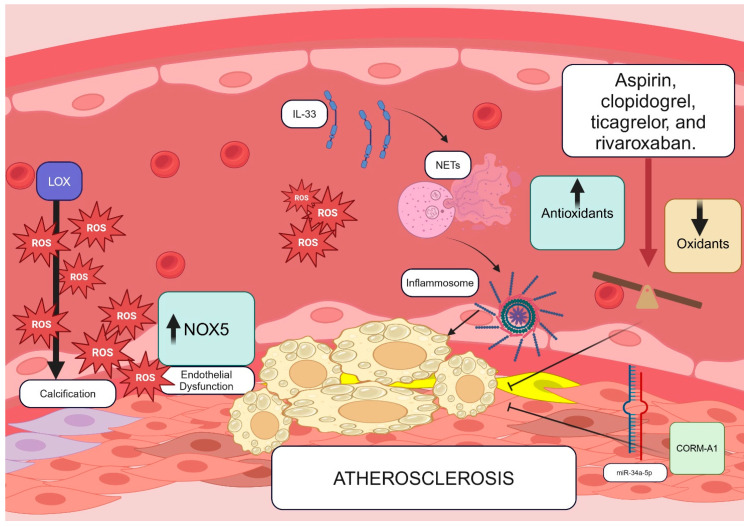
This figure illustrates several potential mechanisms of atherosclerosis discussed in this Special Issue. These include the upregulation of LOX-1, which promotes the calcification of the extracellular matrix, thereby modulating the process of arterial atherosclerotic calcification. Another mechanism involves the overexpression of NOX-5, leading to endothelial dysfunction through the excessive production of reactive oxygen species (ROS). Additionally, an increased concentration of Interleukin 33 (IL-33) may encourage the formation of neutrophil extracellular traps (NETs), resulting in greater ROS production via the NLRP3 inflammasome pathway. An antioxidant and anti-atherosclerotic effect could potentially be achieved using certain antithrombotic drugs or by activating the CORM-A1 pathway, which is mediated by the inhibition of miR-34a-5p.

This Special Issue evaluated several steps of atherosclerotic damage caused by oxidative stress on the cardiovascular system, including Lysyl oxidase (LOX)-mediated extracellular matrix remodeling, the pathogenic role of IL-33, the potential of NOX5 overexpression to promote endothelial dysfunction, and the anti-atherosclerotic effects of CORM-A1, as well as the antioxidant effects of antiplatelet and anticoagulant therapies (Figure 1). While these mechanisms warrant further investigation in larger human studies, they may represent important avenues for exploring the atherosclerotic process. However, it is important to mention other pathogenic mechanisms of atherosclerosis, such as the role of other NADPH oxidase isoforms, like NOX2 [2] or nitric oxide [16], and other pathways implicated in oxidative stress, such as dysbiosis [17], which are fundamental but were not covered in this Special Issue.

Regarding the antioxidant effects of anticoagulant and antiplatelet therapies [18,19], it remains unclear whether these effects can effectively mitigate oxidative stress in atherosclerosis and its complications. So far, intervention studies involving vitamin supplementation have yielded mixed results in terms of reducing cardiovascular complications. Furthermore, the role of antioxidants in preventing the onset and recurrence of arrhythmias such as atrial fibrillation is still unclear [20]. Therefore, future prospective, randomized controlled trials are needed to assess the clinical impact of these therapies.
